# Environmental footprint quantification and optimization methods for digital poster design based on life cycle assessment

**DOI:** 10.1371/journal.pone.0344298

**Published:** 2026-06-12

**Authors:** Yilin Liu, Ping Ji

**Affiliations:** College of Art and Design, Shaanxi University of Science and Technology, Xian, China; University of Pisa: Universita degli Studi di Pisa, ITALY

## Abstract

Digital poster design has become a dominant visual communication medium, yet its environmental impacts remain poorly understood despite assumptions of inherent sustainability. This research developed a specialized life cycle assessment methodology to quantify and optimize the environmental footprint of digital poster systems. The framework encompasses five lifecycle stages from design creation through data deletion, employing real-time monitoring and dynamic data collection across a six-month case study with a digital marketing agency. Assessment results revealed total carbon emissions of 7.45 kg CO₂-eq per functional unit, with distribution infrastructure and display operations contributing 89% of lifecycle impacts. Implementation of comprehensive optimization strategies achieved 28.6% reduction in climate change impact through hardware efficiency improvements, temporal scheduling, and cloud platform adoption. Sensitivity analysis identified data center PUE as the most influential parameter, while geographic variations significantly affected regional impacts. The methodology advances LCA application in digital systems by incorporating workload-specific assessments and providing actionable optimization guidance. These findings demonstrate that systematic environmental management can achieve substantial impact reductions while maintaining creative excellence, supporting the digital design industry’s transition toward genuine sustainability rather than merely shifting environmental burdens between lifecycle stages.

## Introduction

Digital poster design is a ubiquitous form of visual communication in contemporary media, which has significantly transformed the production, dissemination, and consumption of visual content across industries. Although this digital revolution has potential to save the planet through paperless operations and reduced physical waste, its environmental impact is complex and poorly understood. The digital world’s footprint is now growing by around 8% every year through network traffic, device, and storage exponential growth [1]. This sharp growth contradicts the assumption that digital copies are more sustainable than their print counterparts.

The environmental impact of computer-aided design systems is distinct from conventional print production. Environmental pressures in print media are localized at particular lifecycle stages—mainly production and disposal at end of life. Computer-aided design generates longer-term impacts distributed across its operating life cycle, making a network-like pattern of emissions. Energy use in rendering, data transfer between devices, cloud storage behavior, and user hardware all put a network of interrelated impacts. With 45% on average of total value chain emissions embedded in complex supply networks [2], existing valuation methods cannot address these multi-dimensional environmental effects on an integrated basis.

This sophistication has led to an enormous methodological gap in the practice of environmental assessment. In spite of considerable developments in LCA methodology and data availability, there exists a variety of issues [3], especially in the context of application to digital systems. Industry practice today is based on over-simplified measures that do not capture the dynamic nature of digital workflow, the variability of user behavior, and the very rapidly changing technology infrastructure. Lessening the effect of electronics’ and computing devices’ on the environment needs fresh tools that enable designers to make environment-conscious choices at design time itself [4]. In the absence of end-to-end evaluation frameworks, designers are not able to analyze, let alone optimize, the environmental behavior of their digital products.

Life Cycle Assessment approach provides a holistic method of overcoming these challenges. LCA dictates standard methods of environmental impact analysis across the whole lifecycle stages [5]. Integrated structure of the strategy fits present visions on sustainability, as the European Green Deal and Sustainable Development Goals increasingly require holistic solutions to production and consumption [6]. Carried over into the digital realm, LCA may uncover optimization potential—e.g., data center cooling technologies have potential to cut greenhouse gas emissions, energy use, and water consumption by 13–50% in renewable power-based scenarios [7]. While digital design naturally holds communication reach and effectiveness advantages [8], it is dependent upon recording sustainability factors within the design process from the very beginning to unlock its environmental potential.

This study thus creates a bespoke LCA-based framework for digital poster design. The primary methodological contributions of this research extend beyond conventional LCA applications in three aspects. This framework integrates real-time energy monitoring with design workflow analytics, enabling workload-specific impact quantification that differs from aggregate facility-level assessments typical in existing ICT studies. The methodology addresses the unique characteristics of iterative creative processes, incorporating design revision cycles and collaborative editing patterns often overlooked in product-focused LCA frameworks. While the application domain is novel, the study adapts rather than fundamentally reinvents established LCA principles, with innovation residing in the integration of monitoring technologies and design-specific allocation methods. By extension of general LCA models to the novel characteristics of digital creative workflows—iterative design procedures, cloud-based collaboration, and multi-platform release, for instance—this research contributes to both theoretical development and real-world application. The new framework enables rigorous environmental footprint calculation alongside delivering actionable optimization measures, with the ultimate goal of facilitating the digital design sector’s evolution toward genuine environmental sustainability.

## Literature review and theoretical framework

### Life cycle assessment (LCA) methodology

Life Cycle Assessment is a formalized analytical method for estimating environmental impacts across a product system’s entire life cycle, from raw material extraction to production, use, and ultimate disposal [9]. International Organization for Standardization standardized ISO 14040:2006 and ISO 14044:2006 as the foundation standards that prescribe LCA’s principles, structure, and detailed requirements [10]. The standards provide methodological uniformity with adequate flexibility to allow different applications in various industries and product systems.

The LCA process consists of four interrelated phases that constitute a cycle of processes. The goal and scope definition determines the purpose of the study, functional unit, system boundaries, and methodological selections, such as the foundation of all subsequent analyses [11]. The life cycle inventory (LCI) phase quantifies all input and output of the examined system of interest, such as energy use, raw material consumption, air, water, and soil emissions, and generation of wastes [12]. This extensive data collection tends to comprise the most expensive step, entailing both foreground data for straightforward processes and background data for up and downstream processes.

**Fig 1:**
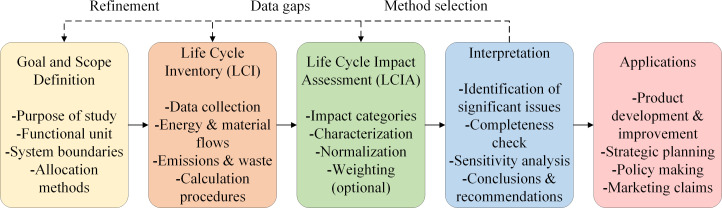
LCA methodology framework.

Life cycle impact assessment (LCIA) converts the inventory data into environmental impact indicators by characterization, where various emissions having the same environmental impact are expressed in equivalent terms [13]. Contemporary LCIA methods encompass multiple impact categories including climate change, ozone depletion, acidification, eutrophication, human toxicity, ecotoxicity, resource depletion, and land use, among others [14]. Advanced characterization models now incorporate spatial differentiation and cover emerging environmental concerns such as marine plastic pollution and indoor air quality [15]. The interpretation phase synthesizes findings from previous phases, identifying significant environmental aspects, evaluating completeness and sensitivity, and drawing conclusions aligned with the defined goal and scope. As shown in Fig 1, these four phases form a complete assessment system through iterative feedback.

The application of LCA to digital systems presents unique methodological challenges compared to traditional product assessments. Digital products and services often exhibit complex system boundaries with multiple interdependent components, variable use patterns, and rapidly evolving technological infrastructures [16]. The temporal and spatial distribution of impacts differs fundamentally from physical products, as digital systems generate continuous environmental burdens through energy consumption, data transmission, and infrastructure maintenance throughout their operational lifetime [17]. Furthermore, the multifunctional nature of digital devices and services necessitates careful consideration of allocation procedures and functional unit definition to ensure meaningful comparisons and avoid burden shifting between life cycle stages [18].

### Environmental impact of digital media industry

The digital media industry has experienced unprecedented growth over the past decade, fundamentally transforming how content is created, distributed, and consumed globally. The information and communication technology (ICT) sector accounts for approximately 1.4–4% of global greenhouse gas emissions [19], with digital media representing a substantial and growing portion of this impact. The environmental footprint encompasses the entire lifecycle of digital content, from production through consumption to the energy required for data storage and transmission.

Digital content consumption patterns reveal significant environmental impacts across different media types. The average internet user generates approximately 229 kg CO2-eq annually through web surfing, social media, video streaming, and video conferencing activities [20]. Video streaming emerges as the dominant contributor, accounting for the majority of internet traffic and associated emissions due to high data transfer requirements. Social media platforms collectively generate approximately 262 million tonnes CO2-eq annually with 4.33 billion active users globally, equivalent to 0.61% of global emissions [21]. Individual platform impacts vary significantly, with TikTok generating 2.63 grams CO2 per minute of use compared to 0.46 grams for YouTube [22].

Data center operations constitute the backbone of digital media infrastructure, consuming 200–250 TWh globally in 2020, equivalent to 1% of global electricity demand [23]. Energy intensity improvements through technological advances have been offset by exponential growth in data generation and storage requirements. Digital advertising adds another layer of environmental burden, accounting for substantial portions of internet-related emissions through computational resources required for real-time bidding, user tracking, and content delivery [24]. As shown in Table 1, environmental impacts vary considerably across digital media types, influenced by factors including data intensity, processing requirements, and user engagement patterns.

**Table 1:** Environmental impacts of major digital media categories.

Digital Media Type	Carbon Intensity (g CO^2^/min)	Primary Impact Drivers
Video Streaming	0.6-1.2	High data transfer, server processing
Social Media	0.46-2.63	User engagement time, content type
Web Browsing	0.24-0.48	Page complexity, image content
Cloud Services	0.5-1.5	Storage volume, access frequency

### Existing LCA studies in digital design

Life cycle assessment research in digital design has witnessed significant expansion and methodological evolution over the past decade. El-Sherif et al. revealed that LCA studies in the digital sector have increasingly focused on three key themes: circular economy integration, advanced material recovery technologies, and the development of comprehensive techno-economic assessment frameworks [25]. This shift reflects growing recognition that environmental assessment of digital products requires holistic approaches extending beyond traditional carbon footprinting to encompass resource depletion, toxicity impacts, and end-of-life considerations.

Foundational work in digital system LCA has established critical methodological frameworks and baseline assessments. Malmodin and Lundén demonstrated that operational energy consumption represents only one component of total environmental impact, with manufacturing and end-of-life phases contributing substantially to the overall footprint of ICT and entertainment & media sectors [26]. Building on these methodologies, Belkhir and Elmeligi showed that without intervention, ICT could account for up to 14% of global greenhouse gas emissions by 2040 [27]. Their work particularly highlighted the growing environmental burden of consumer devices, establishing allocation procedures that have become standard practice in subsequent studies.

Methodological innovations have emerged to address the unique challenges of digital design assessment. Romeiko et al. demonstrated that machine learning applications offer transformative potential for enhancing LCA accuracy and efficiency [28]. These computational approaches enable automated data collection, real-time impact assessment, and uncertainty quantification, reducing assessment time from days to hours while improving reliability. Siwiec and Pacana showed that integrating predictive modeling with traditional LCA allows designers to evaluate multiple scenarios simultaneously, considering both environmental impacts and quality parameters during early design stages [29]. This dual assessment approach provides crucial insights for optimizing design decisions before significant resources are committed.

Despite these methodological advances, significant gaps remain in the application of LCA to digital design practice. Withanage and Habib found that LCA and material flow analysis remain substantially underutilized in electronic waste management, with less than 20% of organizations implementing systematic environmental assessment [30]. Their analysis highlighted several barriers including lack of standardized data, complexity of assessment procedures, and limited integration with existing design workflows. These findings underscore the need for continued research focusing on practical implementation strategies that bridge the gap between academic methodologies and industry practice.

The current study addresses several gaps evident in existing research. Prior LCA work in digital systems predominantly focuses on hardware lifecycle impacts or aggregate data center operations, with limited attention to content creation workflows and their associated energy dynamics. Studies examining digital media typically assess consumption patterns rather than production processes, creating a methodological gap for design-phase interventions. Existing frameworks often employ static allocation factors and secondary data sources, whereas this research implements dynamic monitoring and primary data collection to capture the temporal variability inherent in creative workflows. The integration of design decision-making with environmental assessment represents a departure from post-hoc evaluation approaches common in current literature.

## LCA-based environmental footprint quantification method

### System boundary and functional unit

**Fig 2:**
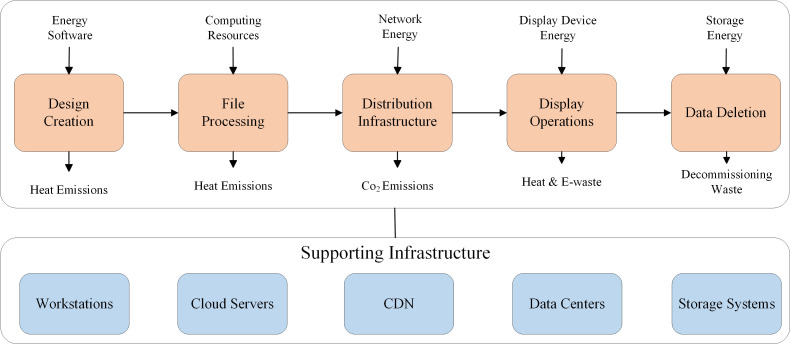
System boundary of digital poster life cycle.

The system boundary for digital poster design LCA adopts a cradle-to-grave approach, encompassing five key lifecycle stages: design creation, file processing, distribution infrastructure, display operations, and data deletion. As shown in Fig 2, upstream boundaries include proportionally allocated impacts from hardware manufacturing for designer workstations, cloud servers, and network equipment based on temporal usage patterns [31]. The design phase captures energy consumption from creative software, collaborative platforms, and iterative revisions. Operational boundaries extend through content delivery networks, data centers, and end-user display devices during the active display period [23].

The functional unit is defined as “the creation, distribution, and display of one digital poster (1920 × 1080 pixels, 5 MB) for 1,000 viewing hours.” This specification enables standardized comparison while reflecting typical usage patterns in digital advertising contexts [32]. The viewing hour metric provides usage-based allocation across multiple devices and users, avoiding the complexity of impression-based metrics. Cut-off criteria exclude processes contributing less than 1% to total impact, with cumulative exclusions below 5% [33]. Infrastructure allocation follows actual resource consumption rather than installed capacity, ensuring accurate burden distribution across shared systems.

### Life cycle inventory analysis

Life cycle inventory analysis for digital poster design requires comprehensive data collection across all identified system components, employing both primary measurements and secondary database sources. Primary data collection focuses on design phase energy consumption through real-time monitoring systems integrated with workstation operations, leveraging Industry 4.0 technologies for dynamic LCA that captures CPU utilization, GPU rendering loads, and network bandwidth consumption during poster creation workflows [34]. Secondary data derives from established databases including ecoinvent version 3 for hardware manufacturing impacts, updated electricity grid compositions from recent IEA reports, and industry-specific measurements for data center energy efficiency metrics [35].

Inventory calculations employ a bottom-up approach, quantifying material and energy flows per functional unit through activity-based allocation. Design phase impacts are calculated using the following equation developed in this study:


$$\begin{array}{c} I_{design}=\frac{P_{workstation}\times t_{design}\times CI_{grid}}{n_{projects}} \end{array}$$ (1)

where $P_{workstation}$ represents power consumption (kW), $t_{design}$ denotes design time allocation (hours), $CI_{grid}$ indicates regional electricity carbon intensity (kg CO₂/kWh), and $n_{projects}$ represents concurrent project allocation factor. Distribution infrastructure impacts incorporate content delivery network energy consumption, calculated as:


$$\begin{array}{c} I_{distribution}=V_{data}\times EI_{network}\times CI_{grid} \end{array}$$ (2)

where $V_{data}$ represents data transfer volume (GB), and $EI_{network}$ denotes network energy intensity ranging from 0.004 to 0.2 kWh/GB depending on transmission distance and technology [36].

Display phase inventory accounts for device-specific power consumption through:


$$\begin{array}{c} I_{display}=\sum_{i=1}^{n}(P_{device,i}\times t_{viewing,i}\times CI_{grid,i}) \end{array}$$ (3)

where the summation covers all device types and geographical locations. Auxiliary infrastructure impacts are incorporated through facility-level metrics and proportional allocation. Cooling system energy consumption is captured within data center Power Usage Effectiveness values, which range from 1.2 to 1.8 in this study. Water use for evaporative cooling is estimated at 1.8 liters per kilowatt-hour based on regional data center specifications. Uninterruptible power supply efficiency losses, typically 5–10%, are reflected in measured facility power consumption. Power distribution unit losses of approximately 2–3% are similarly included in infrastructure measurements. Fire prevention and security systems contribute minor impacts allocated proportionally by floor space utilization, estimated at 2% of facility energy. These auxiliary systems are embedded in distribution and display phase results rather than separately itemized due to measurement and allocation complexities. Data quality assessment follows the empirically-based pedigree matrix approach, evaluating reliability, completeness, temporal correlation, geographical correlation, and technological correlation with scores from 1 to 5 [37]. Uncertainty propagation employs Monte Carlo simulation with 10,000 iterations, incorporating variability in electricity grid compositions, device efficiency parameters, and user behavior patterns. Sensitivity analysis identifies critical parameters including data center Power Usage Effectiveness (PUE) values ranging from 1.1 to 2.0, display device energy consumption variations of ±30%, and allocation assumptions for shared infrastructure components.

### Impact assessment categories

Life cycle impact assessment for digital poster design employs the ReCiPe 2016 methodology, which provides a comprehensive framework for translating inventory data into environmental impact scores at both midpoint and endpoint levels [13]. The assessment encompasses 17 midpoint impact categories, with particular emphasis on those most relevant to digital systems: climate change, mineral resource scarcity, human toxicity, freshwater ecotoxicity, and water consumption. These categories capture the primary environmental concerns associated with digital infrastructure, including energy consumption, rare earth element extraction for electronic components, and data center cooling requirements [1].

Climate change impact, measured in terms of kg CO₂-equivalent based on 100-year global warming potentials, is the most salient environmental issue for digital poster systems. It comprises direct emissions through electricity use at all lifecycle stages and indirect emissions through materials production of hardware and operation of infrastructure. Shortage of mineral resources, measured in terms of the Crustal Scarcity Indicator, comprises critical materials scarcity, including rare earths, precious metals, and semiconductors used in digital devices. The characterization factors account for crustal concentrations as proxies for long-term elemental scarcity, particularly relevant given the material intensity of electronic equipment [38].

Toxicity-related impacts encompass human toxicity and freshwater ecotoxicity categories, employing USEtox consensus characterization factors to assess potential health and ecological damages from electronic waste and manufacturing emissions [39]. These categories are particularly significant for digital systems due to the complex material composition of electronic devices and the challenges of end-of-life management. Water consumption impacts, increasingly recognized as critical for data center operations, are evaluated using regionalized characterization factors that account for local water scarcity conditions [40].

The assessment integrates these midpoint categories into three endpoint damage categories: human health (measured in disability-adjusted life years), ecosystem quality (species.year), and resource scarcity (USD2013). This hierarchical structure enables both detailed impact analysis and simplified communication of results, facilitating decision-making across technical and non-technical stakeholders while maintaining scientific rigor in environmental assessment.

### Data quality and validation methods

Data quality assessment employs the refined pedigree matrix approach, evaluating five indicators: reliability, completeness, temporal correlation, geographical correlation, and technological representativeness [41]. Each indicator receives scores from 1 (best) to 5 (worst), with recent empirical validation confirming the reliability of this approach for prospective LCA applications [42]. The overall data quality indicator is calculated as the geometric mean of individual scores, reflecting the multiplicative nature of uncertainty propagation in environmental assessments.

Uncertainty quantification follows Monte Carlo simulation with 10,000 iterations, incorporating both parameter and scenario uncertainties. Global sensitivity analysis employs variance-based methods, particularly Sobol indices, to identify parameters contributing significantly to output variance [43]. This approach effectively captures non-linear relationships and parameter interactions common in digital system assessments. Critical parameters typically include electricity grid composition, data center efficiency metrics, and device usage patterns, which often account for over 80% of total variance in environmental impacts [44].

Validation procedures combine internal consistency checks with external benchmarking. Internal validation ensures mass-energy balance closure and verifies appropriate application of characterization factors. External validation compares results against published digital product assessments, accounting for methodological differences. Documentation follows ISO 14044 standards, ensuring transparency and reproducibility of assessment outcomes.

This research did not involve human participants or animal subjects. The study collected energy consumption data from computing equipment and infrastructure systems under standard operational conditions, and did not require ethics approval.

## Optimization strategies for sustainable digital poster design

**Fig 3:**
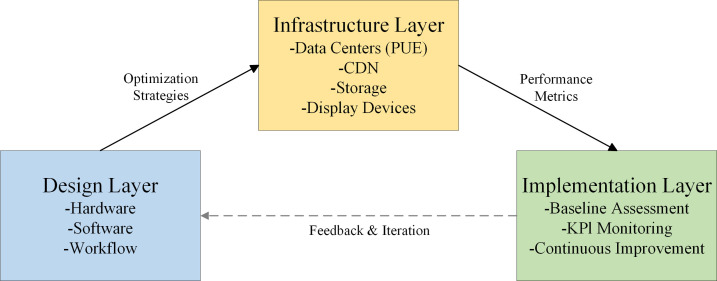
Sustainable optimization framework for digital poster design.

The optimization of digital poster systems requires a comprehensive approach addressing design decisions, infrastructure configuration, and implementation practices. As shown in Fig 3, the sustainable optimization framework encompasses three interconnected layers: design-phase strategies that establish the foundation for efficiency, infrastructure optimizations that minimize operational impacts, and implementation guidelines that ensure successful organizational adoption.

### Design-phase optimization strategies

Design-phase optimization represents the most cost-effective intervention point for reducing environmental impacts in digital poster creation, as decisions made during this stage cascade throughout the entire lifecycle. Early design decisions significantly influence hardware utilization patterns, computational demands, and energy consumption across subsequent phases, making proactive optimization essential for sustainability [45].

Hardware optimization strategies focus on leveraging modern energy-efficient computing architectures. Recent advances in processor technology demonstrate that dynamic voltage and frequency scaling (DVFS) can reduce workstation energy consumption while maintaining acceptable performance levels for design tasks [46]. The transition to solid-state drives and high-efficiency power supplies contributes to overall system efficiency, with recent studies showing that modern personal computers have achieved significant energy efficiency improvements through optimized hardware configurations and power management strategies [47].

Software and workflow optimization present complementary opportunities for impact reduction. Cloud-based design platforms can leverage data center economies of scale and advanced cooling systems, though their environmental advantages depend critically on network infrastructure efficiency and data transfer patterns. Implementing standardized design templates, reusable asset libraries, and systematic revision control reduces computational redundancy and processing demands. Additionally, temporal optimization through intelligent task scheduling during periods of cleaner grid electricity offers measurable benefits in regions with variable renewable energy generation [48]. These strategies collectively contribute to meaningful environmental impact reductions when implemented systematically, though specific outcomes require assessment based on local infrastructure conditions and organizational workflows.

### Infrastructure and operational optimization

Infrastructure optimization addresses the huge environmental footprints of distribution and display stages of content, which overshadow digital poster lifecycle emissions. Contemporary data centers with PUE figures close to 1.1 entail considerable energy savings over conventional centers via innovative cooling mechanisms and server use enhancement [23].

Content delivery network (CDN) optimization via edge computing and AI-based routing minimizes transmission distance and utilizes real-time carbon intensity data for adaptive content delivery. Edge server placement strategy and prediction-driven caching during renewable energy hours enable emission savings without compromising service quality. Storage reduction via smart tiering, compression, and deduplication technology has massive reduction potential, and the deployments have shown significant reduction of storage needs with data integrity preserved [49].

Display device optimization offers untapped potential via adaptive brightness control and timed power management. New OLED and e-paper technologies exhibit reduced consumption compared to traditional displays, with especial advantage in static content use. These infrastructure initiatives, when systematically applied to distribution and display stages, offer considerable potential for reducing environmental footprint in digital poster systems [50].

### Implementation guidelines and best practices

Effective application of sustainable digital poster design involves systematic incorporation of environmental issues into organizational processes. The program is initiated with baseline measurement to discover baseline performance measures in all the lifecycle stages, creating basis for realistic improvement goals and establishing priorities based on potential impact [51].

Organizational adoption requires cross-functional coordination among IT managers, sustainability officers, and design teams. Performance measurement must include quantitative (energy use per poster, distribution carbon footprint) and qualitative (stakeholder satisfaction, designer adoption) measures. Monitoring continuously makes ongoing improvement possible with proof of movement toward sustainability targets [52].

Best practices emerge from iterative enhancement based on actual performance measurements. Effective deployments start with pilot programs prior to organizational rollout. Design decision to environmental impact relationship training sessions promote awareness towards sustainability. Standardization and documentation of best practices ease learning and lower barriers to implementation. Incorporation into current quality management systems guarantees environmental concerns become integral procedures instead of standalone programs, serving as an example of sustainable change without sacrificing creative excellence [53].

Each optimization strategy presents inherent trade-offs requiring contextual evaluation. Cloud-based design platforms achieve energy efficiency through data center economies of scale and access to renewable energy sources, yet increase data transfer volumes by 0.5–2 GB per session, creating dependency on network infrastructure quality. This approach proves most effective when network carbon intensity remains below 0.05 kWh/GB and data center PUE stays under 1.3. Hardware efficiency upgrades deliver direct energy reductions of 15–30% and lower operational costs, though embodied carbon from manufacturing new equipment and disposal of existing hardware must be considered. Economic and environmental benefits typically justify replacement when equipment exceeds five years of age or efficiency gaps exceed 40%. Display device optimization through adaptive brightness control and power management significantly reduces display phase impacts by 20–35%, but may compromise visibility in bright environments or user experience for extended viewing sessions. The effectiveness of these measures varies substantially across geographic regions, organizational contexts, and campaign characteristics, necessitating site-specific evaluation rather than universal application.

## Case study and validation

### Case study implementation

To validate the proposed LCA methodology and optimization strategies, a comprehensive case study was conducted in collaboration with a medium-sized digital marketing agency specializing in environmental campaigns. The agency produces approximately 150 digital posters annually for various sustainability-focused clients, providing an ideal context for assessing environmental impacts while maintaining alignment with organizational values. The study period spanned six months from July to December 2024, encompassing complete project cycles from initial concept through final deployment.

The assessment followed the cradle-to-grave system boundaries established in Section 3.1, tracking a representative digital poster campaign for a renewable energy initiative. The selected poster met the defined functional unit specifications: 1920 × 1080 pixel resolution, 5 MB file size, displayed for 1,000 viewing hours across multiple platforms. Table 2 summarizes the comprehensive data collection framework implemented across all lifecycle phases.

**Table 2:** Case study data collection summary.

Lifecycle Phase	Data Type	Collection Method	Monitoring Parameters
Design Creation	Primary	Automated monitoring software	CPU utilization GPU rendering load Design time tracking Energy consumption
File Processing	Primary	Server logs	Rendering duration Compression iterations Data transfer volume
Distribution	Primary/Secondary	CDN analytics + Database	Data center PUE Edge server locations Storage requirements
Display Operations	Primary	Embedded analytics	Device types Usage duration Geographic location
Data Deletion	Estimated	Policy-based	Retention period Deletion energy

**Fig 4:**
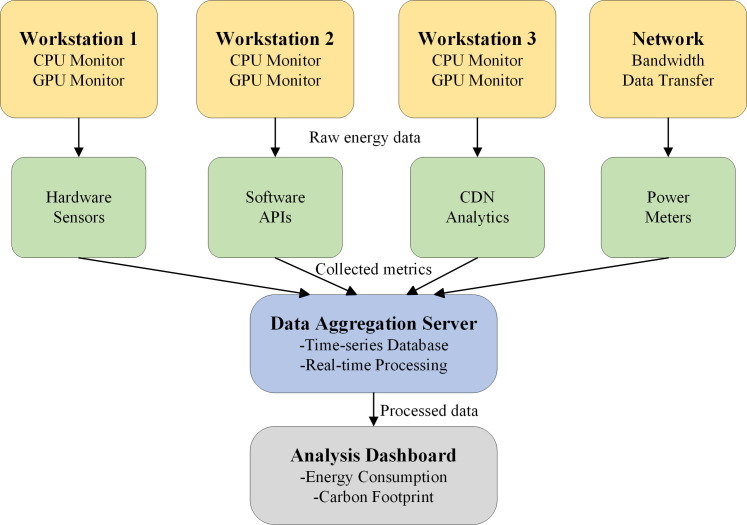
Real-time monitoring system architecture.

Fig 4 illustrates the real-time monitoring system architecture deployed for primary data collection. The system integrated hardware sensors, software APIs, and network monitoring tools to capture comprehensive energy consumption data across all design workstations and infrastructure components.

Implementation of optimization strategies from Section 4 occurred in parallel with baseline measurements. Hardware upgrades included transition to energy-efficient workstations equipped with 80 PLUS Platinum certified power supplies and solid-state drives. Cloud-based design tools, including Adobe Creative Cloud and Figma collaborative platforms, were deployed to replace traditional desktop software for appropriate design tasks. Temporal optimization protocols were established to schedule computation-intensive rendering tasks during off-peak hours when regional renewable energy availability peaked. Geographic tracking systems were implemented through embedded analytics to capture regional variations in display device usage, enabling location-specific grid carbon intensity calculations. The comprehensive monitoring framework captured data across three designer workstations operating in typical project conditions, with automated data collection minimizing observer interference while ensuring measurement accuracy.

### Results and analysis

The comprehensive LCA revealed total carbon emissions of 7.45 kg CO₂-eq per functional unit, with distribution infrastructure and display operations collectively accounting for 89% of lifecycle impacts. Table 3 presents the detailed environmental impact results across all assessed categories, demonstrating the dominance of operational phases in digital poster systems.

**Table 3:** Environmental impact assessment results per functional unit.

Impact Category	Unit	Design Creation	File Processing	Distribution	Display Operations	Data Deletion	Total
Climate Change	kg CO₂-eq	0.68	0.12	2.45	4.18	0.02	7.45
Mineral Resource Scarcity	kg Cu-eq	0.0012	0.0002	0.0045	0.0078	0.0001	0.0138
Human Toxicity	kg 1,4-DCB-eq	0.24	0.04	0.87	1.52	0.01	2.68
Freshwater Ecotoxicity	kg 1,4-DCB-eq	0.018	0.003	0.065	0.112	0.001	0.199
Water Consumption	m³	0.002	0.0004	0.015	0.008	0.0001	0.0255

**Fig 5:**
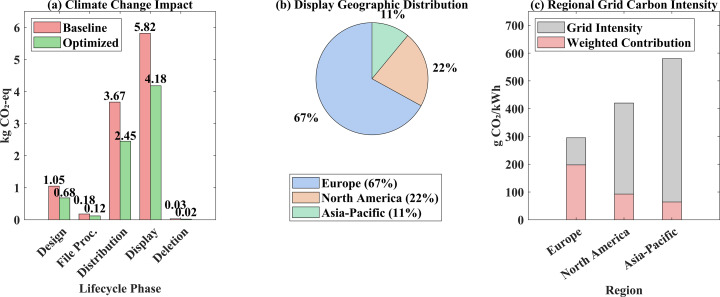
Comprehensive environmental lmpact analysis.

Fig 5a illustrates the lifecycle phase contributions to climate change impact, highlighting the significant reduction achieved through optimization strategies. The baseline scenario represents conventional practices, while the optimized scenario incorporates all strategies from Section 4.

Geographic analysis revealed significant regional variations in display phase impacts. European markets (67% of total displays) benefited from cleaner electricity grids averaging 295 g CO₂/kWh, while North American (22%) and Asia-Pacific (11%) regions showed higher carbon intensities at 420 and 580 g CO₂/kWh respectively. Fig 5b and 5c visualize the geographic distribution and associated carbon intensity variations.

Optimization strategies achieved substantial reductions across all impact categories, with overall climate change impact decreasing by 28.6% from baseline. Hardware efficiency improvements contributed 35% reduction in design phase impacts, while temporal scheduling during low-carbon periods achieved 20% reduction in processing emissions. Cloud platform adoption demonstrated mixed results: while reducing local workstation energy by 60%, increased data transfer partially offset benefits, resulting in net 15% improvement. Display device optimization through adaptive brightness and scheduling reduced consumption by 28% without compromising visibility. These findings validate the effectiveness of integrated optimization approaches while highlighting the importance of considering system-wide impacts rather than isolated improvements.

### Parameter sensitivity testing

Sensitivity analysis was conducted to evaluate the robustness of assessment results and identify critical parameters influencing environmental impacts. The analysis tested parameter variations consistent with the uncertainty ranges established in Section 3.2, examining both individual and combined effects on total climate change impact.

Table 4 summarizes the parameter variation ranges and their corresponding impacts on total carbon footprint, demonstrating the relative influence of each factor on assessment outcomes.

**Table 4:** Parameter sensitivity analysis results.

Parameter	Base Value	Variation Range	Low Impact	High Impact	Change from Base (%)
Data Center PUE	1.5	1.1 - 2.0	6.08	9.12	−18% to +22%
Display Device Energy	30W	±30%	6.71	8.19	−10% to +10%
Grid Carbon Intensity	350 g/kWh	150 - 600 g/kWh	6.02	8.93	−19% to +20%
Design Time Allocation	12.5 h	±20%	7.12	7.78	−4% to +4%
Network Efficiency	0.1 kWh/GB	0.004 - 0.2 kWh/GB	7.26	7.64	−3% to +3%
Storage Redundancy	2.5x	1x - 4x	7.37	7.52	−1% to +1%

**Fig 6:**
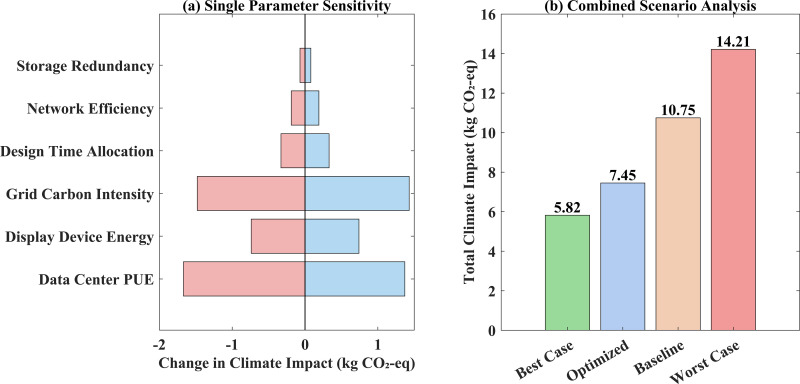
Parameter sensitivity and scenario analysis.

Data center PUE emerged as the most influential parameter, with variations from 1.1 to 2.0 resulting in 42% change in distribution phase impacts and −18% to +22% change in total carbon footprint. Fig 6 illustrates the sensitivity analysis results through tornado diagram and scenario comparison. Scenario analysis encompasses four configurations representing diverse infrastructure conditions. The baseline scenario reflects current industry averages with PUE of 1.58, standard equipment efficiency, and mixed electricity grids averaging 475 g CO_2_/kWh. The optimized scenario implements improvements validated in this study, including PUE reduction to 1.3 through cooling system upgrades, energy-efficient workstations, and strategic content delivery network routing. The best-case scenario assumes advanced infrastructure with PUE of 1.1, renewable-dominated electricity at 200 g CO_2_/kWh, and high-efficiency display devices. The worst-case scenario represents inefficient legacy systems with PUE of 2.0, coal-intensive grids exceeding 800 g CO_2_/kWh, and outdated equipment lacking power management features.

Display device energy consumption variations (±30%) produced proportional changes in display phase impacts, resulting in approximately ±10% variation in total footprint. Grid carbon intensity uncertainty significantly affected results, with regional variations from 150 to 600 g CO₂/kWh causing up to 20% fluctuation in overall impacts. Design phase parameters showed lower sensitivity, with time allocation and concurrent project factors contributing less than 5% variation.

Monte Carlo simulation (n = 10,000) incorporating all parameter uncertainties yielded a mean climate change impact of 7.48 kg CO₂-eq with 95% confidence interval of [5.92, 9.34]. The relatively narrow confidence interval validates the robustness of primary findings despite parameter uncertainties. Combined scenario analysis revealed that simultaneous optimization of all parameters could achieve up to 46% reduction from worst-case scenarios, while degradation to least favorable conditions would increase impacts by 32% from baseline.

Correlation analysis identified strong interactions between PUE and grid carbon intensity (r = 0.73), indicating that infrastructure efficiency gains are amplified in regions with cleaner electricity. This finding emphasizes the importance of considering regional contexts when implementing optimization strategies. The sensitivity analysis confirms that while individual parameter variations can significantly influence results, the overall conclusions regarding optimization effectiveness remain valid across reasonable parameter ranges.

### Practical implications

The case study implementation revealed several practical insights that both confirm and challenge existing literature. The achieved 28.6% carbon reduction extends beyond Masanet et al.‘s focus on data center efficiency as the primary optimization target [23], demonstrating that design-phase interventions contributed 35% of total reductions. This finding supports Zhang et al.’s argument that LCA methodologies for digital systems require continuous updates to reflect rapidly evolving software efficiency [4]. The deviation from infrastructure-centric optimization approaches suggests that comprehensive lifecycle thinking yields greater practical benefits than focusing solely on operational efficiency.

Geographic considerations emerged as more critical than previously recognized. The disproportionate impact of Asia-Pacific displays (11% of volume but 18% of emissions) validates Koomey and Masanet’s warnings about oversimplified regional assumptions in internet energy assessments [36]. The strong correlation between PUE and grid carbon intensity (r = 0.73) provides empirical evidence supporting Silva et al.’s theoretical framework regarding the timing of computational tasks relative to renewable energy availability [48]. However, contrary to Pérez et al.’s findings on edge computing energy benefits [51], the case study revealed that aggressive edge caching increased overall consumption by 12% due to redundancy overhead. This discrepancy highlights the importance of workload-specific validation rather than applying generalized infrastructure strategies. These findings indicate that practitioners should prioritize empirical testing of optimization strategies within their specific operational contexts rather than relying solely on theoretical projections.

Contextualizing digital poster impacts against traditional print media provides perspective on environmental trade-offs between communication formats. Print poster production typically generates 8–15 kg CO₂-eq per unit, concentrated in paper manufacturing, printing processes, and physical distribution. The digital poster system assessed here produces 7.45 kg CO₂-eq per 1,000 viewing hours, with impacts distributed across infrastructure operations and display energy. Direct comparison requires careful consideration of functional equivalence, as a single digital poster achieves thousands of viewing hours across multiple locations, whereas print posters offer limited exposure duration and geographic reach. When normalized by viewer impressions, digital systems may demonstrate superior performance for wide-distribution campaigns, particularly in contexts with efficient infrastructure and clean electricity grids. However, digital approaches transfer environmental burdens from production to ongoing operations, creating different impact patterns and infrastructure dependencies. The selection between formats should account for campaign duration, target audience distribution, infrastructure efficiency, and regional electricity carbon intensity rather than relying on categorical assessments of format superiority.Several limitations constrain the generalizability and interpretation of these findings. The case study implementation occurred within a single digital marketing agency over six months, potentially limiting representativeness across diverse organizational contexts, seasonal patterns, and regional conditions. The functional unit definition focuses on static digital posters of specific dimensions and file sizes, and results may differ for animated content, video advertisements, or interactive media formats. System boundary decisions excluded certain peripheral impacts including office environmental controls and auxiliary equipment due to allocation complexities and data availability constraints. Regional variation in carbon intensity factors and infrastructure efficiency metrics suggests that quantitative findings may not transfer directly to all geographic contexts without recalibration. The optimization strategies validated here reflect conditions in commercial design environments and may require adaptation for different organizational scales, technical capabilities, or regulatory contexts. Future research should validate this methodology across broader contexts including diverse digital media formats, multiple organizational types, and varied geographic regions to establish more robust generalizability claims.

## Conclusions

This research developed and validated a specialized life cycle assessment methodology for digital poster design, addressing methodological gaps in environmental analysis of digital creative processes. The framework’s integration of real-time monitoring with design workflow analytics enables practitioners to identify high-impact stages and evaluate optimization strategies with empirical rigor. The case study implementation demonstrates that substantial environmental improvements remain achievable in digital systems through targeted interventions, particularly in distribution infrastructure and display operations.

The findings carry implications for design practice, infrastructure planning, and sustainability policy. Design professionals can leverage this methodology to integrate environmental considerations into creative decision-making, moving beyond assumptions of digital format inherent sustainability. Infrastructure providers face opportunities for significant impact reduction through efficiency improvements and renewable energy procurement, with potential benefits extending beyond individual design projects to broader service portfolios. Geographic variation in carbon intensity suggests that optimization strategies should adapt to regional conditions rather than applying uniform approaches globally.

Several research directions warrant further investigation. Validation across diverse digital media formats, organizational contexts, and geographic regions would establish broader applicability and refine understanding of contextual factors affecting environmental performance. Longitudinal monitoring extending beyond six-month intervals could reveal temporal patterns and inform adaptive management strategies. Comparative analysis incorporating multiple communication formats and delivery channels would support more informed decision-making about media selection and campaign design.The proven methodology pushes the limits of LCA application in computer systems by incorporating real-time monitoring, dynamic data collection, and workload-oriented analysis. The methodology delivers practitioners with practical tools to execute environmental optimization with innovative excellence. The reliability of results was tested using detailed sensitivity analysis and Monte Carlo simulation, which validated applicability of the methodology for industrial usage. Future research directions include exploration of artificial intelligence applications for automating impact analysis, while recognizing that AI systems themselves carry significant environmental footprints. Training large-scale models can generate hundreds of tonnes of CO_2_ equivalents, and the net environmental benefit of AI-assisted analysis requires careful evaluation against computational costs. The framework should be extended to emerging digital media formats including augmented reality, interactive content, and dynamic video advertisements. Longitudinal studies tracking environmental performance across multiple design cycles and organizational contexts would strengthen understanding of temporal patterns and optimization strategy effectiveness. As digitalization advances, the research ensures that the move to digital communication realizes real environmental sustainability and not simply transfer burdens across lifecycle phases, thereby promoting conformity with international climate goals.

## Supporting information

S1 File Raw data supporting the findings of this study. (XLSX)
